# Small-Scale Processing of High-Performance BNNT Fiber for Space and Electronics Applications

**DOI:** 10.1021/acsanm.5c03860

**Published:** 2025-11-13

**Authors:** Casey L. Smith, Keenan J. Mintz, Kishor Gupta, Anita Garg, Laura Wilson, Satish Kumar

**Affiliations:** † School of Materials Science and Engineering, 1372 Georgia Institute of Technology, , Atlanta, Georgia 30332, United States; ‡ Department of Mechanical, Industrial and Manufacturing Engineering, University of Toledo, Toledo, Ohio 43606, United States; § 53522 NASA Glenn Research Center; Cleveland, Ohio 44135, United States

**Keywords:** boron nitride nanotubes, BNNT fiber, nanotube orientation, oxidation resistant materials, nanoscale materials processing

## Abstract

A method to process boron nitride nanotube (BNNT) fibers with a high degree of alignment, high modulus, and good tensile strength is presented. This has been achieved by dispersing BNNTs in a polymer solution, spinning the resulting polymer/BNNT dispersion into fibers, and removing the polymer. Significant alignment is imparted to the BNNTs within the fiber during drawing and heat treatment under tension. These BNNT fibers are characterized structurally and elementally to confirm the BNNT structure. This work has resulted in a highly oriented BNNT fiber with a modulus as high as 396 GPa and a tensile strength as high as 500 MPa. These tensile values represent the current state of the art for BNNT fibers, and the alignment of BNNTs in the fiber is the highest ever achieved for nanotubes-based fibers. Significant porosity is observed from the TEM images of the BNNT fibers’ cross section, indicating that further processing optimization can be expected to further increase these properties. A knot can be made in some of the resulting BNNT fibers, suggesting that some of these BNNT fibers are suitable for typical textile processing techniques. BNNT fibers, their textile preforms, and BNNT fiber containing composites will be suitable for applications requiring high thermal conductivity without electrical conductivity, high temperature oxidative resistance, and low dielectric constant, particularly in aerospace and electronics areas.

## Graphical abstract

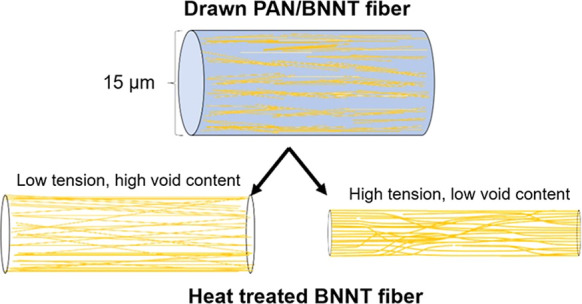

## 1 Introduction

In the last two decades, key innovations have been made in the synthesis and applications of boron nitride nanotubes (BNNTs). BNNTs are electrical insulators with a band gap of 5–6 eV and have oxidative resistance to ∼850 °C,^1^ low-k dielectric properties,^2^ high thermal conductivity,^3^ and high strength.^4^ This combination of properties makes BNNTs ideal for applications requiring one or more of these attributes, particularly for electronics cooling and space exploration. Additionally, several studies have been conducted to remove impurities in BNNTs.^5–7^ Further processing of the BNNTs into fibers and films highly depends on the amount and form of impurities, as well as the alignment that can be imparted to the BNNT network.^8–10^ The thermal, electrical, and mechanical properties of individual BNNTs have driven research into the production of macromaterials of nanotubes, such as fibers and films. For individual BNNTs, tensile strengths and modulus of more than 100 GPa and 0.8 TPa have been predicted, respectively.^11,12^ Experimental studies have shown that the performance of BNNTs is typically lower than this (14–61 GPa).^13^ Experimental modulus values have also widely varied (from 0.7 to 1.2 TPa), but in this case, some values are higher than predicted, which is attributed to increased crystallinity resulting from multiple walls.^14–16^ This considerable degree of variation may affect the performance in yarns and fibers composed of many nanotubes or even small bundles of nanotubes. For CNTs, which also possess different structures and varying alignment and length in each bundle, a decrease has been shown in the net tensile strength of a CNT bundle as the number of included carbon nanotubes increased.^17^ The development of BNNT macrostructures is a nascent technology, and significant progress is needed to improve the mechanical properties of individual nanotubes.

Only two reports of neat BNNT fibers exist to-date: wet spinning of fibers from a solution of BNNTs^18^ and direct formation of fibers from the BNNT plume after synthesis.^19^ The wet spinning report involved forming a lyotropic solution of BNNT in chlorosulfonic acid and resulted in moderately aligned BNNT fibers with an average modulus of 1.5 GPa and an average tensile strength of 16 MPa.^18^ The direct fiber formation method twists BNNTs together from a high-enthalpy plasma method into BNNT yarn or fiber with a modulus of ∼0.5 GPa and a tensile strength of ∼10 MPa.^19^ These fibers produced in the literature have limited commercial application due to their low alignment, strength, and modulus. Some more work has been performed on the polymer/BNNT composite fiber. Lim et al. used polymeric stabilizers to utilize the liquid crystalline properties of BNNTs to form composite fibers with a strength of around 250 MPa.^20^ Kim and co-workers developed a composite fiber with BNNTs dispersed in an aromatic amide-based polymer which showed moderate orientation and a modulus of around 18 GPa, which is a good value for a polymer-based fiber for textile applications.^21^ The improvement in modulus of PVA fibers with inclusion of BNNTs has also been shown, as this led to an increase in modulus of about 13×.^22^ BNNTs have also been studied in polyacrylonitrile (PAN) and carbon fibers.^23^ The presence of BNNTs in these fibers appeared to reduce the activation energies for certain stabilization reactions but did not have a significant effect on the resulting carbon fibers. More recently, the dispersion and purification processes of BNNTs were studied to understand the effect of these parameters on the spinning conditions with the goal of maximizing orientation of the BNNTs in the PAN/BNNT fiber.^24^ In order to realize the full capabilities of BNNTs in aerospace applications, it is necessary to obtain a neat BNNT structure which does not have polymeric material present and can achieve high temperature stability.

In this manuscript, we build upon our previous work^24^ and present a new method to produce BNNT fibers with a high degree of alignment, high modulus, and good tensile strength. This has been achieved by dispersing BNNTs in a polymer solution, dry-jet wet spinning the resulting polymer/BNNT dispersion, and then removing the polymer during tension heat treatment (Scheme 1). These fibers are suitable for various high temperature composites that require high thermal conductivity, high temperature oxidative resistance, and low dielectric constant.

**Scheme 1:**
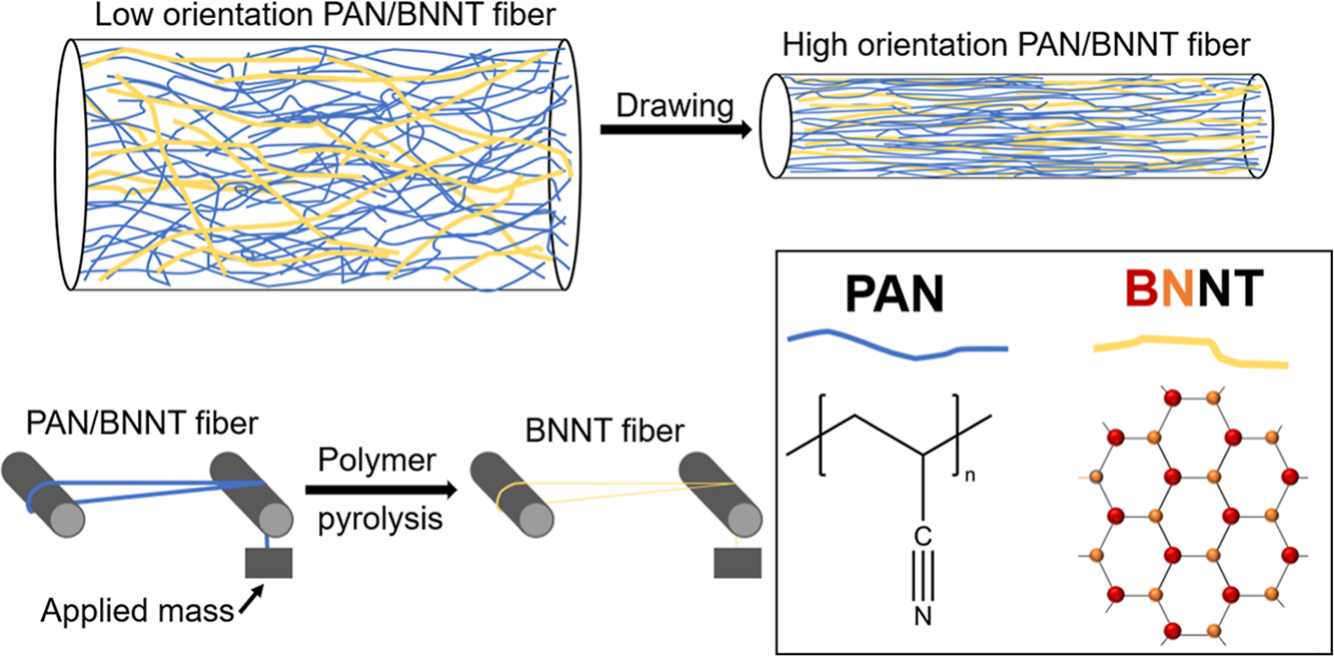
Graphical Depiction of the Drawing and Heat Treatment Process^a^

a PAN and BNNTs are aligned in the drawing process which allows for greater stress to be applied during heat treatment. This stress imparts further orientation and brings the BNNTs into contact with each other upon polymer burnout.

## 2 Experimental Section

### 2.1 Materials

PAN homopolymer, copolymer containing 4 wt % methacrylic acid, and copolymer containing 4% methyl acrylate were obtained from Japan Exlan Co. Poly­(methyl methacrylate) (PMMA) was purchased from Sigma-Aldrich. Boron nitride nanotubes were acquired from BNNT, LLC and are produced with a catalyst-free high temperature/high pressure method and have an average length of 20 μm and a diameter of 3–5 nm. HPLC grade dimethylacetamide (DMAc) was used as received from Sigma-Aldrich as the solvent for both PAN and PMMA polymers. Methanol for the coagulation bath was used as received and was acquired from VWR. Heat treatment in controlled environments of ammonia, air, or nitrogen was performed using an alumina tube furnace and compressed gas cylinders supplied by Airgas.

### 2.2 Precursor Fiber Preparation and Heat Treatment

Polymer/BNNT solutions were prepared as described in our previous work.^24^ Briefly, a polymer solution was prepared by adding polymer powder to a chilled DMAc bath and raising the temperature to 90 °C to dissolve the polymer. This was followed by the addition of a dilute dispersion of BNNTs and evaporation of excess solvent to obtain a spinnable solution. Details of BNNT dispersion/purification and fiber spinning are given in Table S1. Draw ratios referred to in Table S1 and throughout the manuscript are related to the precursor fiber preparation (e.g., fiber spinning and drawing processes). Polymer/BNNT precursor fibers underwent heat treatment to remove the polymer and to further align BNNTs. This was done in a tube furnace under air, nitrogen, ammonia, or a combination of these environments, while maintaining tension (specific environment conditions and stresses are given in Table S2). Tension during heat treatment was between 10 kPa and 50 MPa, depending on the BNNT fiber diameter, number of filaments, and the hanging weight (Table S2).

### 2.3 Characterization

The morphology of the fiber was captured using a Hitachi SU8230 SEM at an accelerating voltage of 5 kV. Elemental composition of fibers was determined using energy-dispersive X-ray spectroscopy (EDS) on fiber cross sections, averaged over 2–3 fibers. Individual fiber cross sections were measured using ImageJ for every trial and at least 10 images were used to calculate the average diameter of the fibers. The tensile properties of the BNNT fiber after heat treatment were determined using a Rheometrics solids analyzer, RSAIII. For the BNNT fiber, tensile tests were conducted at a gauge length of 6 mm and 25.4 mm and at a strain rate of 0.1% per second. Due to the low diameter of fibers in later trials and difficulty separating pristine fibers from the heat-treated tow, fibers with diameter <5 μm were tested in bundles of multiple fibers (50–100) at larger gauge length. The cross-sectional area of the fiber bundles was determined, after tensile testing, using SEM and analyzed by ImageJ. A minimum of 5 samples were tested in each case. Wide Angle X-ray Diffraction (WAXD) of the fiber bundle was carried out using Rigaku Micromax—003 (Cu Kα, λ = 1.542 Å, 60 mA, 50 kV). The small angle scattering signal from the WAXD plate image is also examined. X-ray exposure time was 1–2 h for each sample. The diffraction patterns were analyzed using AreaMax and MDI Jade 9.1 software. The azimuthal Full-Width at Half Maxima (fwhm) was determined from the (200) plane azimuthal scan at 2θ ≈ 26° for BNNTs.

Transmission Electron Microscopy (TEM) was performed at NASA Glenn Research Center after sample preparation using Focus Ion Beam (FIB) milling. TEM foils were FIB milled out from the fibers using a ZEISS Auriga 40 dual focused ion beam FIB-SEM with a Ga ion source. A FEI Talos F200S Scanning/Transmission Electron Microscope was then used for microstructural analysis and high-resolution imaging and characterization. Fourier Transform Infrared Spectroscopy (FTIR) was performed on a Thermo Scientific Nicolet iS5 FTIR Spectrometer in the attenuated total reflectance (ATR) mode. Raman spectroscopy was performed on a Renishaw Raman Spectrometer using 785 nm and a Renishaw inVia UV Raman Microscope at a 266 nm incident laser wavelength.

## 3 Results and Discussion

The addition of polymer to BNNTs offers numerous advantages over the processing of neat nanotubes, including more facile alignment, more resistance to stress, and generally more forgiving processing conditions. While carbon nanotube (CNT) fibers undergo stretching in acid to promote alignment,^25,26^ composite polymer/BNNT fibers can be aligned based on processing techniques that use no acid and employ low temperatures (<200 °C).^27^ However, the polymers used possess significantly lower thermal conductivity and maximum use temperature than those of the individual BNNTs. Since the presence of polymer is not beneficial to the end use of BNNTs as high temperature, thermal management materials, removal of the polymer was targeted while maintaining alignment of the BNNT structure in the precursor fiber (Scheme 1). It is found here that highly oriented BNNT fibers form after the thermal removal of PAN or another polymer in the precursor fiber.

Conventional dry-jet wet spinning techniques were used to form the polymer/BNNT fiber before drawing. Study of the processing, structure, and properties of the polymer/BNNT fiber is detailed in a prior report.^24^ These fibers were then heated under tension to temperatures between 600 and 1100 °C, depending on the polymer and gas environment used. BNNT fibers made from this method show channels of BNNT bundles that travel along the length of the fiber. These channels become visibly straighter and less distorted when progressing from an undrawn precursor under no tension during thermal removal (Figure 1a) to applying tension (Figure 1b and Table S2). Drawing the precursor further refines this process and shows BNNT channels with a much smoother fiber surface (Figure 1c and Table S1). Figure 1d shows the BNNT channel structure, as the fibers experience fibrillation and branch into independent channels.

**Figure 1:**
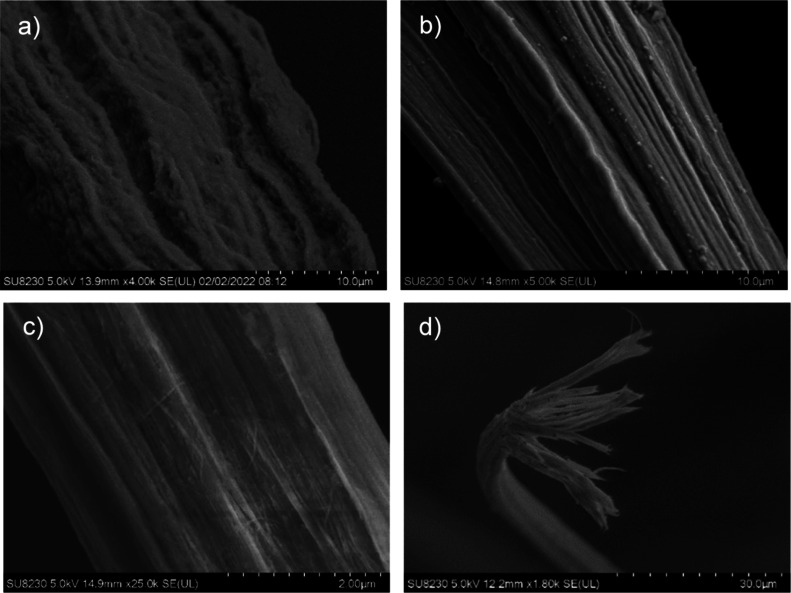
SEM of (a) T1.0-1, (b) T1.0-2, (c) T7.1-2, and (d) T4.2-3 fibers. The transformation between different panels shows the impact of tensioning during heat treatment (a,b) and the impact that draw ratio of the precursor fiber has on the BNNT channels after heat treatment (b,c).

FTIR was performed on BNNT fibers after heat treatment in ammonia (T4.2-5) and air (T7.1-4) and compared to the as-received BNNT puffball (Figure S1). Peaks at ∼800 cm^–1^ and ∼1360 cm^–1^ have been attributed to B–N out-of-plane buckling and in-plane optical phonon modes, respectively.^7^ Burnout of the polymer in an ammonia atmosphere results in purity similar to that of the as-received T-4 BNNT puffball. The burnout is less complete when using an oxygen atmosphere, as a spin finish coating typically used for bundle lubrication remains on the fiber surface and forms a SiO_2_ coating that is readily observed as an additional peak at 1078 cm^–1^. The relative contents of h-BN versus nanotubes in the fibers and puffball are shown in additional FTIR spectra and measurements of peak intensity and area ratios that are provided in Figure S1. Complementary Raman characterization was consistent with the conclusions from FTIR (Figure S2). EDS has also shown that the structure after polymer removal and heat treatment contains boron and nitrogen with few impurities (Table 1).

**Table 1:** Structural Parameters and Tensile Properties of BNNT Fibers from As-Spun Precursors

sample ID	T1.0-1	T1.0-2	T2.0-1	T3.0-1	T4.0-1
precursor draw ratio	2.5	2.5	2.0	2.4	2.0
BNNT content (wt %)	10%	10%	20%	5%	5%
stress during heat treatment (MPa)^a^	none	0.15	0.25	none	0.25
fwhm_(26°)_	37°	23°	40°	25°	32°
crystal size BNNT_(200)_ (nm)	3.1	3.3	3.3	3.4	3.3
equivalent fiber diameter (μm)	19 ± 1.8	15 ± 2.5	16 ± 2.5	21 ± 2.9	12 ± 2.0
tensile strength (MPa) [max]	33 ± 18 [68]	35 ± 18 [72]	30 ± 15 [62]	48 ± 34 [127]	26 ± 18 [32]
modulus (GPa) [max]	4.0 ± 2 [7.7]	12 ± 5 [24]	7.5 ± 5.3 [19]	7 ± 4 [16]	2.8 ± 1.7 [7.1]

a Heat treatment stress was calculated based on the mass attached to the fiber and the BNNT final fiber cross-sectional area.

Table S2 summarizes the heat treatment procedures used for different fiber samples. TX.Y-Z fiber codes are used to represent various processing changes. *X* refers to the trial number, representing different dispersion conditions (Table S1). *Y* refers to the degree of precursor drawing within a particular trial, with *Y* = 0 representing undrawn fiber. *Z* relates to the heat treatment sequence, and generally, increasing values represent higher heat treatment stress within a particular trial. Heat treatment of as-spun fibers without drawing of the precursor led to an average modulus of 12 GPa, showing moderate orientation from the azimuthal scan (fwhm = 23°) and minor elongation during heat treatment under tension (Table 1). When the polymer matrix was changed to poly­(methyl methacrylate) (T3.0-1), as-spun fibers showed the highest tensile strength at 48 MPa with similar orientation to other undrawn fibers (fwhm = 25°, Table 1). These represent early, but successful, attempts to make BNNT fibers using this method. Without drawing or significant heat treatment tension, polymer removal still results in moderately aligned BNNT fibers that roughly match the performance of the existing literature state of the art for BNNT fibers.^18^ The main advantage of this method lies in the ability to further process the polymer fibers before heat treatment and the formation of the BNNT fiber. Drawing, twisting, and other textile operations can be performed with ease on the polymer/BNNT fiber before further heat treatment and creation of a BNNT structure. Due to the favorable heat treatment profile of PAN compared to PMMA (PMMA melts before decomposing which can cause fibers to fuse in the heat treatment process) as well as more favorable spinnability, PAN was chosen as the focus for subsequent studies.

Improvements in the processing of BNNTs at the dispersion stage were made starting from trial T7 (Table S1). This involved centrifuging the samples to eliminate smaller more defected tubes and increasing sonication time to improve the dispersion, which in turn allowed for better spinning properties (i.e., fewer breaks, higher draw ratios).^24^ While sonication helps to improve the dispersion, care must also be given in order to not degrade the tubes in this process.^28^ Significant BNNT orientation can be achieved through drawing the precursor and also by tensioning the fibers during heat treatment. Application of higher tension during heat treatment has a few benefits as noted in Table 2. First, high tension helps maximize BNNT orientation with the fiber axis during polymer heat treatment and reduces shrinkage along the fiber axis (T4.1-2 to T10.2-1). Second, tension reduces the fiber diameter and controls the fiber density. Although tension acts along the fiber axis, the positive Poisson’s ratio of the polymer fiber results in a force that acts along the fiber axial direction. This force drives the densification of the BNNT bundles during thermal removal of the polymer. This behavior is evident in Table 2 as fibers under higher tension have smaller diameters and less shrinkage.

**Table 2:** Structural Parameters and Tensile Properties of BNNT Fibers from Drawn Precursor Fibers

sample ID	T4.1-2	T4.2-3	T4.2-4	T4.2-5	T7.1-1	T7.1-2	T7.1-3	T7.1-4	T7.1-4	T10.2-1
test gauge length	6 mm	6 mm	6 mm	6 mm	6 mm	6 mm	6 mm	6 mm	25.4 mm	25.4 mm
total draw ratio	10	20	20	20	20	20	20	20	20	25
heat treatment gas	NH_3_	NH_3_	NH_3_	NH_3_	air	air	air	air	air	air
stress during heat treatment (MPa)^a^	0.35	1.4	2.8	8.1	9.5	17	50	S1:20	S1:20	S1:20
								S2:30	S2:30	S2:35
shrinkage (%)^b^	–10%	–6.7%	–9.7%	–1.6%	0%	0%	0%	0%	0%	0%
fwhm_(26°)_	19°	19°	16°	11°	9.4°	7.7°	6.9°	6.6°	6.6°	4.8°
crystal size BNNT_(200)_ (nm)	3.7	3.2	3.0	3.7	3.2	3.5	3.7	3.8	3.8	4.6
equivalent fiber diameter (μm)	7.3 ± 1.2	4.4 ± 0.3	3.6 ± 0.6	2.7 ± 0.2	4.0 ± 0.3	3.4 ± 0.2	3.0 ± 0.2	2.7 ± 0.2	2.7 ± 0.2	1.8 ± 0.1
tensile strength (MPa) [max]	32 ± 10 [47]	107 ± 35 [171]	132 ± 21 [220]	153 ± 55 [216]	161 ± 28 [220]	204 ± 28 [255]	271 ± 36 [330]	516 ± 71 [651]	262 ± 24 [296]	498 ± 74 [579]
modulus (GPa) [max]	8.5 ± 3.0 [15]	9.0 ± 2.6 [15]	16 ± 5.0 [26]	55 ± 22 [79]	88 ± 17 [111]	118 ± 15 [202]	184 ± 34 [223]	289 ± 23 [313]	327 ± 28 [352]	378 ± 14 [396]

a Heat treatment stress for trials T4.1-2 to T7.1-3 was calculated based on the mass attached to the fiber and the final BNNT fiber cross-sectional area. S1 and S2 for trials T7.1-4 and T10.2-1 represent two-stage tension, S1 is calculated based on the mass attached to the fiber and initial precursor fiber cross-sectional area while S2 is calculated based on the mass attached and the final BNNT fiber cross-sectional area.

b Shrinkage is the change in length during heat treatment.

Dramatic improvement in the modulus and orientation also occurred once tension during heat treatment was split into two steps (Table 2, T7.1-4 and T10.2-1). Compared to the single-step procedure, a much higher tension is first applied to the fiber at lower temperature by using a 50–80 g mass. The second step then uses the smaller 1–3 g mass when the final, small-diameter BNNT fiber is being formed. Due to the significant change in diameter of the final fiber, stress during the second step of heat treatment is calculated based on the final fiber diameter to give a more accurate estimate for the heat treatment stress contributing to BNNT fiber formation.

As the temperature increases during treatment and PAN is removed by the environment gas, the ability of the fiber to carry the applied tension changes dramatically. Stress increases exponentially as the radius of the fiber decreases from ∼10 to ∼1 μm, showing the BNNTs becoming increasingly responsible for the strength of the fiber. The heating rate and, therefore, the rate at which the polymer is removed is an important factor to increase performance (Table S2). With a lower heating rate, the tensile performance of the final BNNT fiber increases substantially. The slower PAN removal increases the time that tension acts on the BNNTs before they move together and densify into a more oriented structure.

The effects of processing parameters on the fiber structure were studied using WAXD. The molecular orientation within the fiber is studied using the fwhm of the azimuthal scan at 2θ ∼26°, which is characteristic of the BNNT (200) plane. Since the (200) plane is oriented parallel to the fiber axis, a more concentrated signal around the equatorial plane indicates higher orientation. All trials show peaks around 2θ = 26° and 2θ = 42° after heat treatment, corresponding to BN (200) and (100) planes,^29^ respectively. Figure 2 shows the WAXD images of undrawn T4.0 and drawn T4.1 and T7.1 fibers before (a, c, and e) and after (b, d, and f) heat treatment. Drawing of the PAN/BNNT precursor fiber results in a substantial increase in the performance of the BNNT fiber and the upper limit of tension applied during heat treatment. A highly oriented network of BNNTs with improved tensile properties was only possible after drawing the precursor and the application of higher tension with lower heating rates.

**Figure 2:**
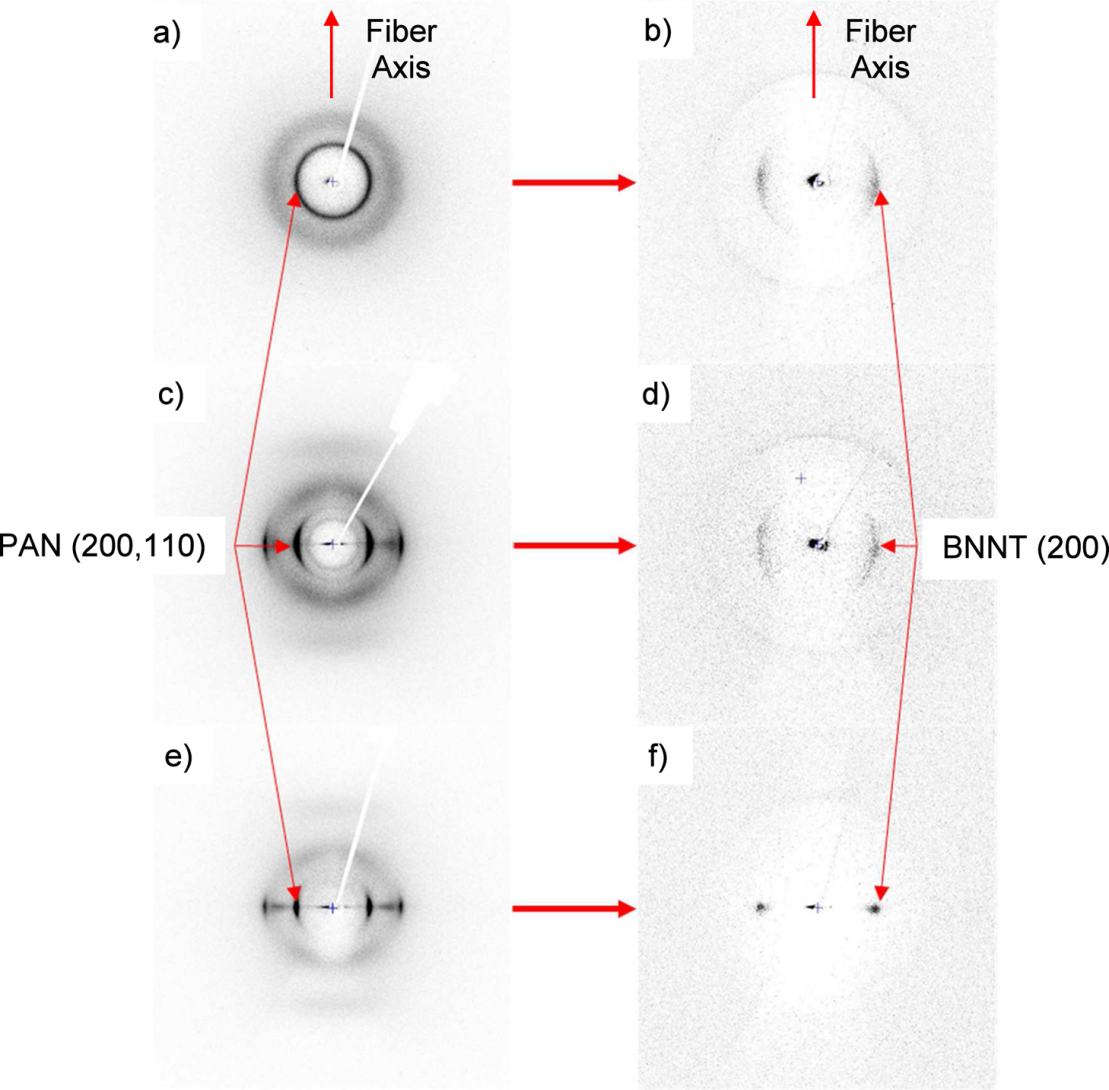
WAXD flat plate photographs of (a) T4.0, (b) T4.0-1, (c) T4.1, (d) T4.1-2, (e) T7.1, and (f) T7.1-3 fibers. The patterns on the left correspond to precursor PAN/BNNT fibers with varying draw ratios (DR = 2 for (a), 10 for (c), and 20 for (e)) and the patterns on the right show the corresponding BNNT fiber after polymer burnout.

The WAXD of the T7.1-3 BNNT fiber shows a very high degree of orientation with a fwhm of the azimuthal scan of 6.9° (Figure 3f). The plate images in Figure S3a–h show the trend of higher orientation of BNNTs along the fiber axis from the concentration of the diffraction rings closer to the equatorial plane at 2θ = 26°. With the application of 50 MPa of tension in trial T7.1-3 (Table 2), the diffraction image is reduced to two dots on the equatorial plane, indicating a very high degree of BNNT orientation on the fiber axis. The alignment reaches its observed maximum for sample T10.2-1, which utilized the two-step heat treatment process (Table 2). The fwhm of T10.2-1 diffraction dots decreases to 4.8° after the application of 20 and 35 MPa of stress during a two-stage heat treatment on the highest draw ratio PAN/BNNT fiber (Table 2).

**Figure 3:**
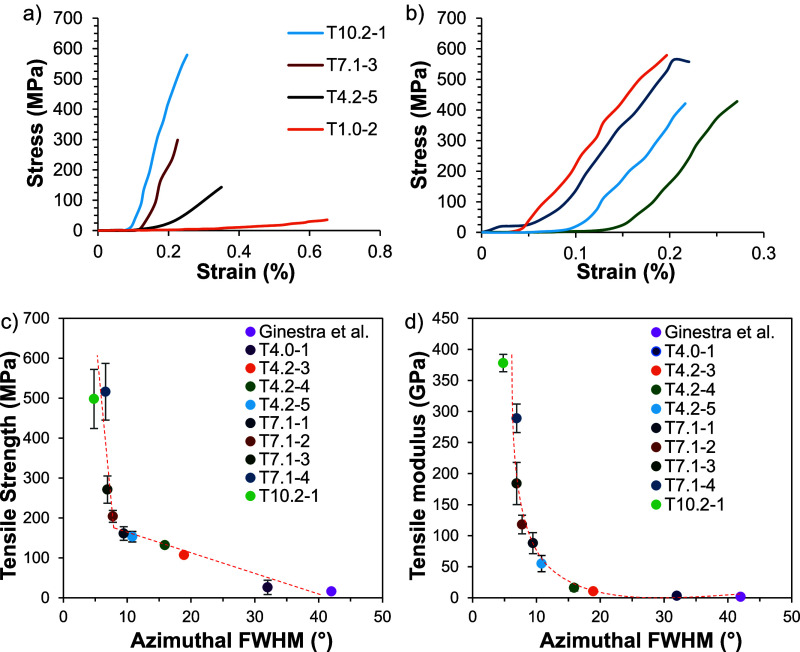
(a) BNNT fiber stress–strain curves demonstrating how fiber strength and modulus increase with increasing draw ratio and tension during heat treatment, (b) T10.2-1 stress–strain samples, and BNNT fiber (c) tensile strength and (d) tensile modulus as a function of fwhm of the azimuthal scan of the ∼26° BNNT WAXD peak.

BNNT orientation is directly related to the performance of the fiber, and there is a strong relationship between high orientation of BNNTs with the fiber axis and high modulus BNNT fibers. This is because the van der Waals forces and weak dipole coupling between tubes are what provide the strength of the fiber and the increased orientation allows for greater surface contact between adjacent tubes which generates stiffness and strength. The increased draw ratio in the precursor stage imparts significant orientation to the nanotubes in the fiber, but stress during heat treatment also plays a significant role as the tubes must be pulled into contact with each other as the polymer is being removed. Trials after T4.2-4 used higher heat treatment tension and a heating rate of 0.5 °C/min, showing an increase in tensile properties from ∼100 MPa strength and <30 GPa modulus to ∼500 MPa strength and ∼300 GPa modulus at a 6 mm gauge length. Figure 3a shows the relationship between the modulus and strength increase on the stress–strain curve from higher orientations, and Figure 3b shows the stress–strain behavior of the highest-performance T10.2-1 BNNT fibers.

The fwhm of the azimuthal scans decreases to <10°, and the highest orientation has a fwhm of 4.8°, strength of ∼500 MPa, and modulus of ∼380 GPa at a 25.4 mm gauge length (Table 2 and Figure 3). Compared to Simonsen Ginestra et al., which recorded an average tensile modulus of 1.46 GPa at an azimuthal fwhm of 42°,^18^ these fibers have significantly higher alignment. Also, Figure 3c,d shows the relationship between the orientation of BNNTs within the fiber, quantified by the WAXD fwhm, and the fiber strength and modulus. The exhibited trend is consistent with other work showing that the substantial increase in the fiber modulus typically starts as the fwhm goes below 10°.^30^

To date, an observed azimuthal fwhm of 4.8° is the highest aligned nanotube fiber ever produced,^31,32^ but the strength of these fibers is only 500 MPa (Table 2). This is significantly lower than other highly aligned nanotube fibers, as CNT fibers have been able to reach 2.5 GPa strength with a fwhm of 6.3°.^33^ Additionally, an increase in the gauge length from 6 to 25.4 mm showed half the recorded strength (T7.1-4, Table 2). These results indicate that the internal pore structure of the fibers plays an important role in the strength outcome. Lowering the heating rate to 0.5 °C/min produced the best outcomes, but there is still room to optimize the process to produce high-strength fibers efficiently.

Increasing the BNNT alignment also leads to a narrowing of the inter-BNNT void space. This inference is observed in the change in the scattering signal at small angles, which can indicate a change in the fiber pore aspect ratio.^34,35^ Figure S4 shows the small angle scattering signal from the plate image of T4.0-1, T4.2-5, and T10.2-1 fibers. This small angle scattering pattern was taken from the plate image of the fibers when the detector distance was calibrated for diffraction. With higher drawing and tensioning in T10.2-1 fibers, this small angle scattering pattern turns into a narrow streak along the equatorial plane that follows narrowing of the space between nanotubes and pore elongation along the fiber axis. The fibrillation of BNNT fibers (Figure 1d) also indicates the presence of well-dispersed, high aspect ratio voids between fibrils.

Significant porosity is still found in the fiber, which does lead to lower-than-expected BNNT fiber strength at the observed alignment. Figure 4 shows cross-sectional TEM images of T10.2-1 fibers after FIB milling to ∼100 Å to produce thin samples for imaging the internal structure of the fiber. Porosity of the T10.2-1 fiber was calculated from ImageJ analysis of TEM images to be around 10 ± 5% of the total area of the fiber cross-section. Additionally, the pore size distribution is quite broad. Small pores with equivalent diameters in the range of 42 ± 8 nm represent most of the pore area. However, the large pores with equivalent diameters in the range of 78 ± 10 nm have the most negative contribution toward the fiber strength.

**Figure 4:**
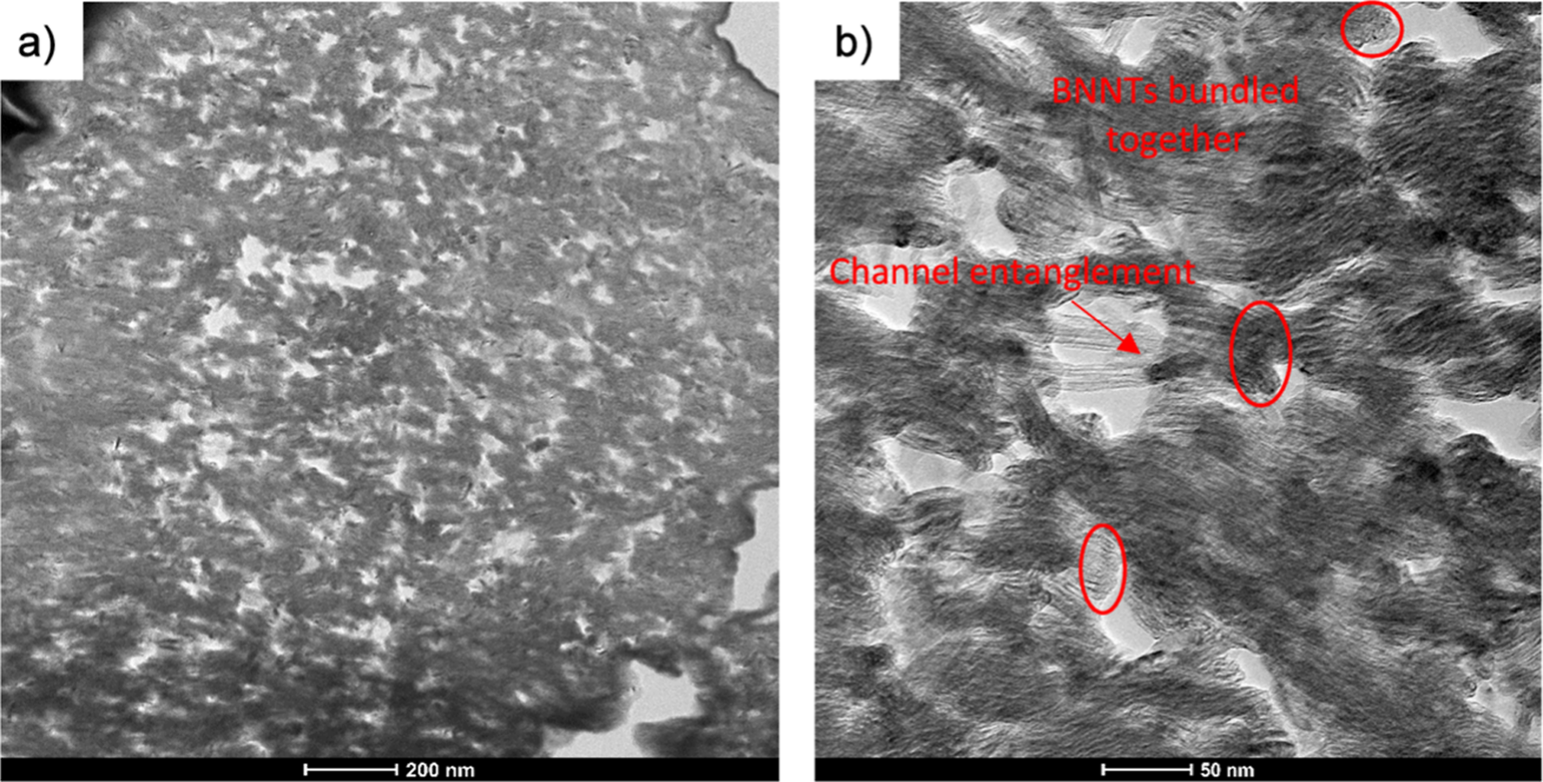
TEM of T10.2-1 fiber cross-section at (a) low and (b) high magnification.

Strategies to reduce these pores mainly include increasing tension, reducing the heating rate, and more completely removing polymer under tension. Fibers produced after T10 struggled to hold more than 50 MPa stress during heat treatment, so it can confidently be said that the highest heat treatment stress for these fibers was achieved. Lowering the heating rate below 0.5 °C/min is possible, but this may decrease the viability of this processing method at scale.

Pores are expected to act as defect points through introducing voids as well as complicating the arrangement of nanotubes within the fiber. It is expected that minor porosity also develops due to inter-BNNT misalignment and entanglement with adjacent nanotubes. At the molecular level, each end of the molecules comprising the fiber will result in a defect size estimated by double the interacting diameter.^36^ Therefore, the BNNTs used here that have a diameter of 3–5 nm will have a defect size from ends of ∼6–10 nm. Additionally, entangled nanotubes magnify this defect size. A defect size of 18–60 nm would be created from 3 to 6 entangled nanotubes. In the observed fiber structure, BNNTs do not exist as independent units but in channels of >10 parallel tubes (Figure 5). Longitudinal TEM of the polymer/BNNT fiber does not show these channels of BNNTs entangling within the polymer fiber.^24^ Channel entanglements are present in the BNNT fiber and most likely develop during polymer removal. Small BNNT channels have equivalent diameters of 20 ± 3 nm, while larger BNNT channels are 31 ± 4 nm in diameter. The channel diameters are around half the small and large pore sizes observed (small pores: 42 ± 8 nm; large pores: 78 ± 10 nm). This observation supports the idea that the BNNT channel entanglements are responsible for the observed defects and pores in the fiber. This could explain the improvement in strength from removing polymer at a lower heating rate: slower polymer removal allows channels more time to arrange without entangling. Regardless, large pores created from entanglements during polymer removal are primarily responsible for the lower strength outcomes of this processing method. Modulus is less affected by these pores, as modulus trends are impacted more by the nanotube orientation, which remains high within the fiber channels.

**Figure 5:**
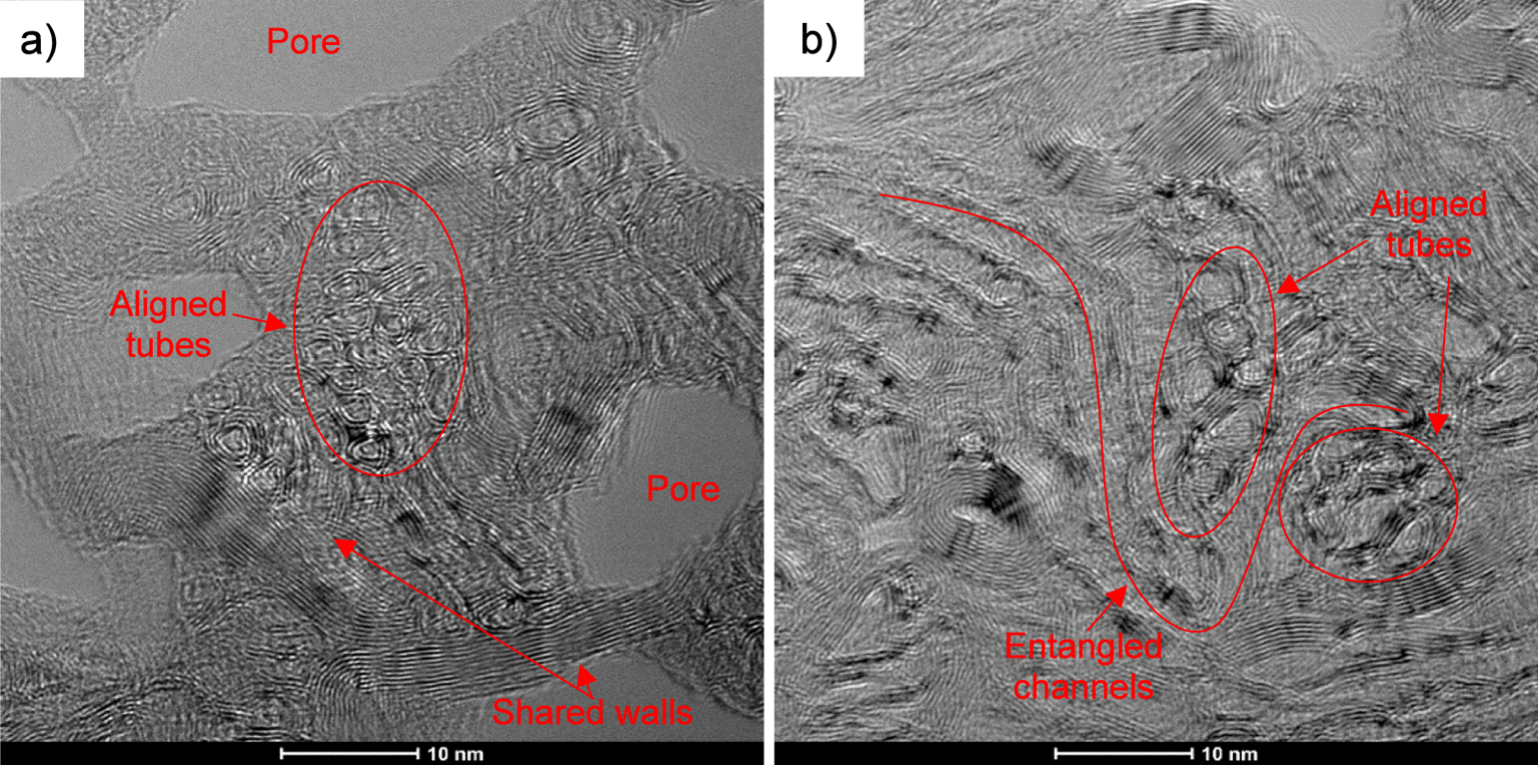
High-magnification TEM of T10.2-1 showing (a) pores and (b) entanglements.

BNNT, LLC reports that the BNNTs used here have 2–5 walls. The most common BNNT appears to be a double-walled tube with a diameter of 3.2 ± 0.6 nm by TEM, though BNNTs with a wall number >8 and diameter of ∼9 nm are also present. Figure 5 shows fiber cross-sectional TEM images of the highly aligned sample, T10.2-1. Figure 5a highlights how aligned nanotube channels coexist with pores. There is also a structure present where multiple nanotubes share a single outer wall. This is consistent with a multi-inner wall nanotube arrangement that forms after heating above 1400 °C.^31^ Additionally, Figure 5b shows how misaligned BNNTs can wrap around BNNTs aligned with the fiber axis to create entanglements.

During drawing of the polymer fiber and heat treatment under tension, the fiber diameter decreases. BNNT channels that are suspended in the fiber near the surface may become entangled with other BNNT channels as the diameter decreases. This sheath of BNNTs acts like a fisherman’s net being pulled toward the fiber core, collecting more BNNTs as polymer is removed and densifying the BNNT network along the sheath. Figure 6 shows a schematic of this behavior. Using a slower heating rate would entail that the net collapses more slowly, giving BNNTs more time to orient with the fiber axis. Increasing the tension during heat treatment gives more driving force for alignment of the BNNTs and densifies the fiber by pulling the net tighter toward the core. BNNT alignment is the most important factor for the fiber properties, as the chemical forces between tubes are what provide the strength of the fiber, and the increased orientation allows for greater surface contact between adjacent tubes.

**Figure 6:**
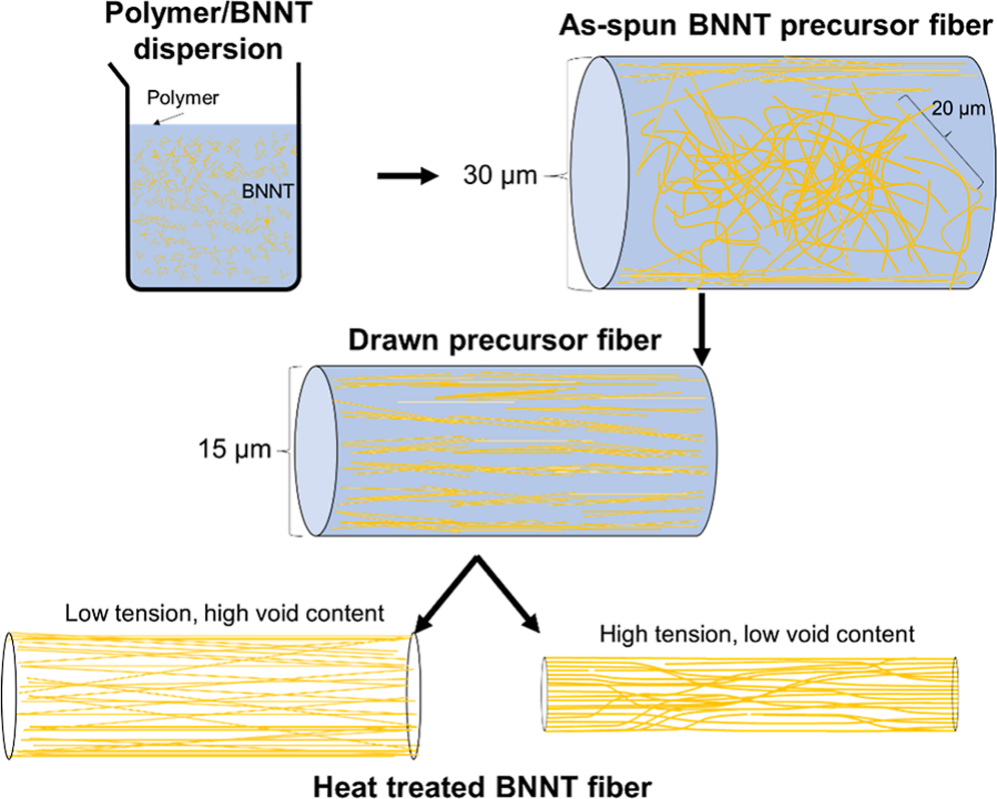
Schematic of polymer/BNNT processing into BNNT fiber.

Due to the polymer burnout mechanism, a sheath-core structure arises after polymer removal with this fiber production process. The core porosity is higher than the sheath porosity, with a greater number of large pores located near the center of the fiber (Figure 4a). This structure likely results from the final core burnout occurring after the fiber sheath is mostly densified. The sheath-core structure and BNNT alignment may be partially responsible for the flexibility and knotting ability of the BNNT fiber. For some fibers, a knot can be tied with the tow, highlighting the mechanical robustness of these fibers (Figure 7). Figure 7a shows fibers that have crimped upon tying a knot, indicating partial collapse of the core at one point along the fiber while the stiff sheath integrity is maintained. These BNNT fibers can be arranged with conventional textile techniques (weaving, braiding, and knitting), opening up applications in composite structures requiring good dielectric strength, oxidation resistance, and high thermal conductivity without electrical conductivity.

**Figure 7:**
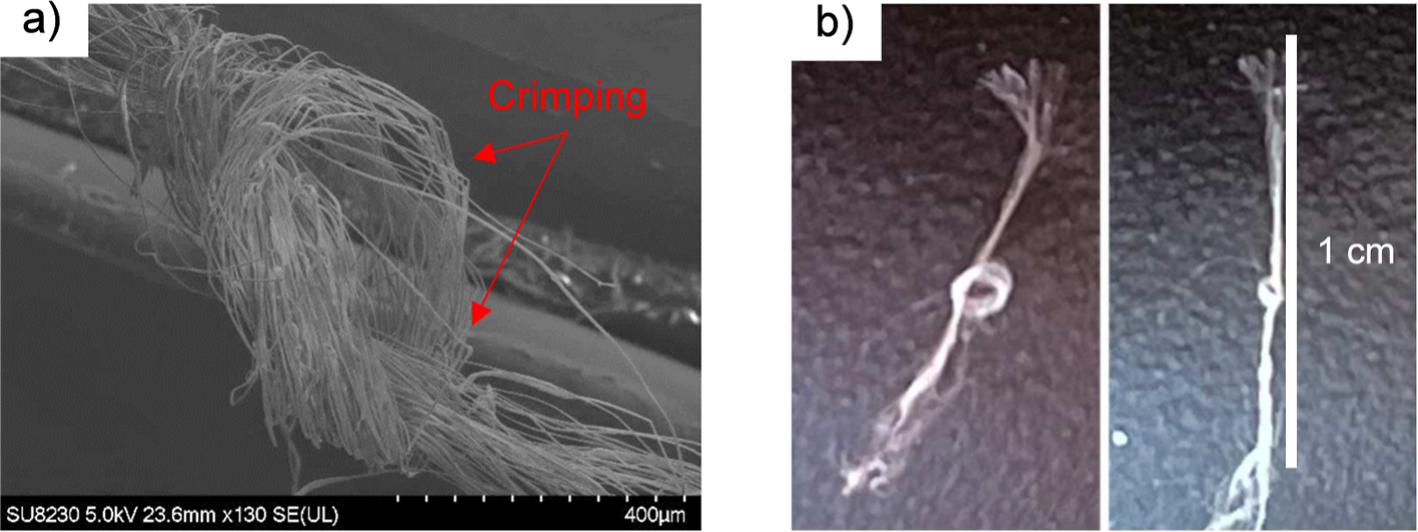
(a,b) Example of a knot in T4.2-4 BNNT fiber.

## 4 Conclusions and Further Work

High orientation BNNT fibers have been produced with a fwhm of 4.8°, strength of 498 ± 74 MPa, and modulus of 378 ± 14 GPa, tested at a 25.4 mm gauge length. This has been achieved by dispersing BNNTs in a polymer solution, spinning the resulting polymer/BNNT dispersion, drawing the as-spun fibers, and then heating the drawn fiber to remove the polymer. These BNNT fibers are characterized structurally and elementally to confirm the BNNT structure. These fibers are the first of their kind with high-performance tensile properties. To the best of our knowledge, these fibers also represent the most highly aligned nanotube fibers produced to date. Work is underway to study the application-specific properties of these fibers, such as thermal conductivity. For CNT fibers of slightly lower alignment (7.9° azimuthal fwhm), thermal conductivity has been observed in the range of ∼350 W/mK.^25^ The high thermal conductivity of nanotube fibers combined with decent strength, high modulus, and electrically insulating properties makes these fibers very good candidates to include in low-k dielectric composites to increase heat management capability. Scale-up production and optimization of drawing, tensioning, and heat treatment processes will also be pursued using tensile and WAXD characterization.

## Supplementary Material



## Data Availability

Data will be made available upon reasonable request.

## Notes

Description of fiber spinning and heat treatment conditions, FTIR and Raman of BNNT fiber samples, and WAXD scans of BNNT fibers (PDF)
