# Supplementary material for: Small-Scale Processing of High-Performance BNNT Fiber for Space and Electronics Applications
Source: ACS Appl Nano Mater. 2025 Nov 13;8(46):22231–40. doi: 10.1021/acsanm.5c03860 (PMC12645434; doi:10.1021/acsanm.5c03860)
Supplement: Supplementary file 1 [file an5c03860_si_001.pdf]

Supporting Information

Small-Scale Processing of High-Performance BNNT Fiber for Space and Electronics Applications

Casey L. Smith[1], Keenan J. Mintz[1*], Kishor Gupta[1], Anita Garg[2,3], Laura Wilson[3], Satish Kumar[1]

1. Georgia Institute of Technology, School of Materials Science and Engineering; Atlanta, GA
2. University of Toledo; Toledo, Ohio
3. NASA Glenn Research Center; Cleveland, Ohio

*Corresponding author email: kmintz3@gatech.edu

Table S1: List of fiber spinning trials (T) and their process conditions.[1]

	**T1**	**T2**	**T3**	**T4**	**T7**	**T9**	**T10**
Polymer matrix	PAN-co-MAA, 500 kg/mol	PAN-co-MAA, 20% 500 kg/mol; 80% 986 kg/mol	PMMA	PAN-co-MAA, 90% 500 kg/mol; 10% 986 kg/mol	PAN, 500 kg/mol	PAN, 250 kg/mol	PAN-co-MA, 510 kg/mol
BNNT (wt% of polymer used)	~10 wt%	~20 wt%	~5 wt%	~5 wt%	~5 wt%	~5 wt%	~5 wt%
Sonication (mins)	30	30	30	30	120	120	40
Dispersion-centrifuge cycle at 5000g	0	0	0	0	1	1	2
As-spun draw ratio	2.5	2	1.2	2	2.3	1.5	2.3
Total draw ratio	2.5	2	2.4	20	20	20	25

Table S2: Heat-treatment conditions for various trials

**Sample**	**Polymer**	**Heat treatment environment**	**Heating rate (°C/min)**	**Heat-treatment Stress (MPa)***	**Heat treatment procedure**	**Fiber stoichiometry**
T1.0-1	PAN	Ammonia	3	none	RT – 1000 °C, 1 hr hold at 1000 °C	$B_{0.9}N_{1.0}C_{0.1}O_{0.06}$
T1.0-2	PAN	Ammonia	3	0.15	RT – 1000 °C, 1 hr hold at 1000 °C	$B_{0.9}N_{1.0}C_{0.2}O_{0.1}$
T2.0-1	PAN	Ammonia	3	0.25	RT – 1000 °C, 1 hr hold at 1000 °C	$B_{0.9}N_{1.0}C_{0.1}O_{0.08}$
T3.0-1	PMMA	Nitrogen	1	none	RT - 350 °C, 15 min hold at 350 °C, 350 – 1000 °C, 1 hr hold at 1000 °C	$B_{0.9}N_{1.0}C_{0.1}O_{0.04}$

T4.0-1	PAN	Ammonia	3	0.25	RT – 1100 °C,<br>1 hr hold at 1100 °C	$B_{0.9}N_{1.0}C_{0.1}O_{0.1}$
T4.1-2	PAN	Ammonia	3	0.35	RT – 1100 °C,<br>1 hr hold at 1100 °C	$B_{0.9}N_{1.0}C_{0.1}O_{0.08}$
T4.2-3	PAN	Ammonia	0.5	1.4	RT – 1100 °C,<br>1 hr hold at 1100 °C	$B_{0.9}N_{1.0}C_{0.1}O_{0.1}$
T4.2-4	PAN	Ammonia	0.5	2.8	RT – 1100 °C,<br>1 hr hold at 1100 °C	-
T4.2-5	PAN	Ammonia	0.5	8.1	RT – 1100 °C,<br>1 hr hold at 1100 °C	$B_{0.9}N_{1.0}C_{0.1}O_{0.1}$
T7.1-1	PAN	Nitrogen; air	0.5	9.5	Step 1 – nitrogen<br>RT – 1500 °C<br>Step 2 – air<br>RT – 700 °C, 30 min hold at 600, 30 min hold at 650 °C; 1 hr hold at 700 °C	$B_{0.9}N_{1.0}C_{0.4}Si_{0.02}O_{0.1}$
T7.1-2	PAN	Air	0.5	17	RT – 700 °C<br>30 min hold at 600, and 30 min hold at 650 °C; 1 hr hold at 700 °C	$B_{1.0}N_{1.0}C_{0.4}Si_{0.05}O_{0.1}$
T7.1-3	PAN	Air	0.5	50	RT – 700 °C<br>30 min hold at 600 °C, 30 min hold at 650 °C; 1 hr hold at 700 °C	$B_{1.0}N_{1.0}C_{0.4}Si_{0.05}O_{0.1}$
T7.1-4	PAN	Air	0.5	Step 1: 20<br>Step 2: 30	Step 1<br>RT – 300 °C<br>Step 2<br>RT – 700 °C<br>30 min hold at 600 °C, 30 min hold at 650 °C; 2 hr hold at 700 °C	$B_{1.0}N_{1.0}C_{0.3}Si_{0.04}O_{0.1}$
T10.2-1	PAN	Air	0.5	Step 1: 20<br>Step 2: 35	Step 1<br>RT – 300 °C<br>Step 2<br>RT – 700 °C<br>1 hr hold at 600; 1 hr hold at 650 °C; 2 hr hold at 700 °C	$B_{1.0}N_{1.0}C_{0.3}Si_{0.07}O_{0.2}$

* Heat treatment stress was calculated based on the hanging weight and the final BNNT fiber cross-sectional area

FTIR was performed on as-received BNNT puffball (T-4), ammonia treated BNNT puffball (T-4 A), and purified BNNT puffball from BNNT, LLC (T-4 P) in Figure S1a. The peak width and peak intensity ratio between the increasing out-of-plane versus in-plane transmission (OP/IP) has been observed to increase as the concentration of h-BN impurity in the sample increases.[2] Therefore, a decrease in h-BN impurity within the puffball or fiber

would lead to a decrease in the intensity or area ratio of the OP/IP ratio. These peak characteristics are listed in Figure S1c. The as-received BNNTs have an OP/IP ratio of 0.75 from intensity and 0.46 from area. The highest OP/IP ratio, indicating the most h-BN impurity, is the T7.1-3 fiber with intensity OP/IP = 1.04. Heat treatment in ammonia to 1100 °C appears to decrease the h-BN content in both puffball and fiber. Other than the purified T-4 P with OP/IP of 0.50 by intensity and 0.21 by area, the T4.2-5 fiber has the 2nd lowest OP/IP ratio of 0.57 by intensity and 0.24 by area.

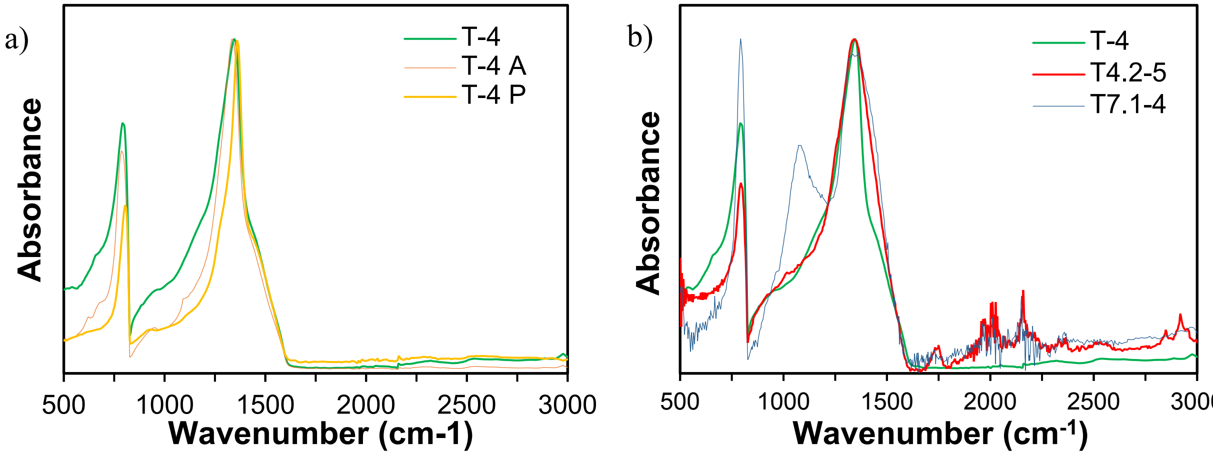

c)

Sample Code	OP/IP (Intensity)	OP/IP (Area)	OP (cm^{-1})	IP (cm^{-1})
T-4	0.75	0.46	795	1346
T-4 A	0.67	0.35	789	1336
T-4 P	0.5	0.21	806	1362
T4.2-5	0.57	0.24	795	1343
T7.1-4	1.04	-	793	1344

Figure S1: FTIR of as-received BNNTs (T-4) compared with (a) ammonia treated BNNT puffball (T-4 A) and purified BNNT puffball (T-4 P); (b) ammonia treated BNNT fibers (T4.2-5) and air treated BNNT fibers (T7.1-4) compared to the as-received T-4 BNNT puffball.; (c) table of OP/IP ratios for the studied samples

Unlike CNTs that have a G and D band, BNNTs have one dominant peak at ~1365 cm^{-1}, corresponding to the E_{2g} mode from the counterphase B-N vibrational mode within the BN sheet. UV Raman on as-received BNNT shows a sharp peak at 1366 cm^{-1} and a decrease in h-BN content is characterized by a broadening in the B-N peak and blue shift in the peak center. T7.1-4 fibers shows a similar sharp BNNT peak upshifted slightly from ~1366 cm^{-1} to ~1369 cm^{-1} and a broad peak around 1600 cm^{-1}. The Raman signals of T4.2-5 and T10.2-1 fibers also show sharp peaks centered ~1369 cm^{-1}, but no broad peaks at 1600 cm^{-1}. The broad peak at 1600 cm^{-1} in T7.1-4 is likely carbon from incomplete PAN removal. The UV Raman signal of puffball and fiber shown in figure S3b and S3c, respectively, also show a noticeable peak around 820 cm^{-1} with shoulder around 795 cm^{-1}. This peak is less prevalent in ammonia treated samples T-4 A and T4.2-5 and is most likely attributed to a boron oxide peak that's blue shifted. Boron oxide will also nitride in ammonia environment.

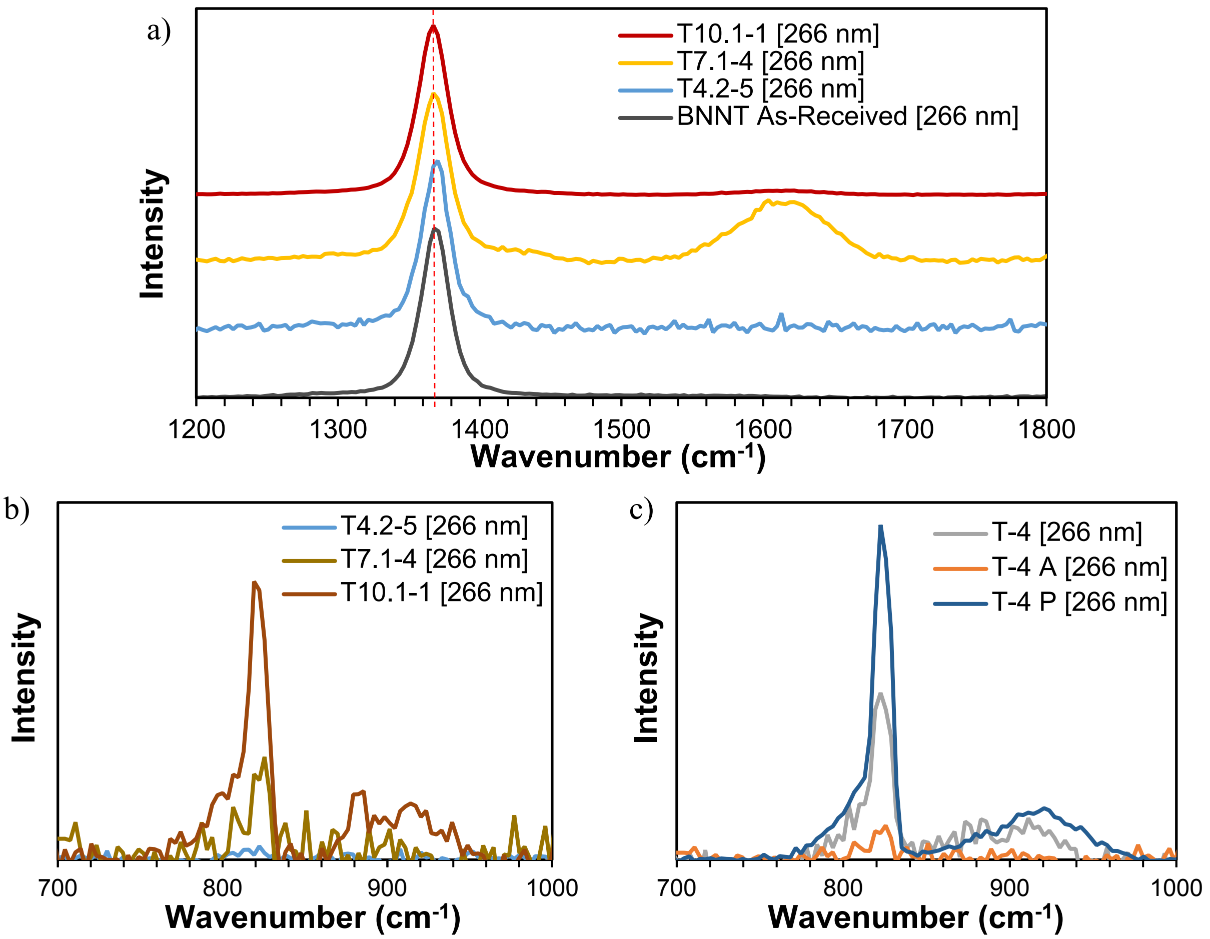

Figure S2: UV Raman spectra of (a) fiber samples showing B-N peak and impurity, (b) fiber samples showing B-O peak presence, and (c) BNNT puffball samples showing B-O peak

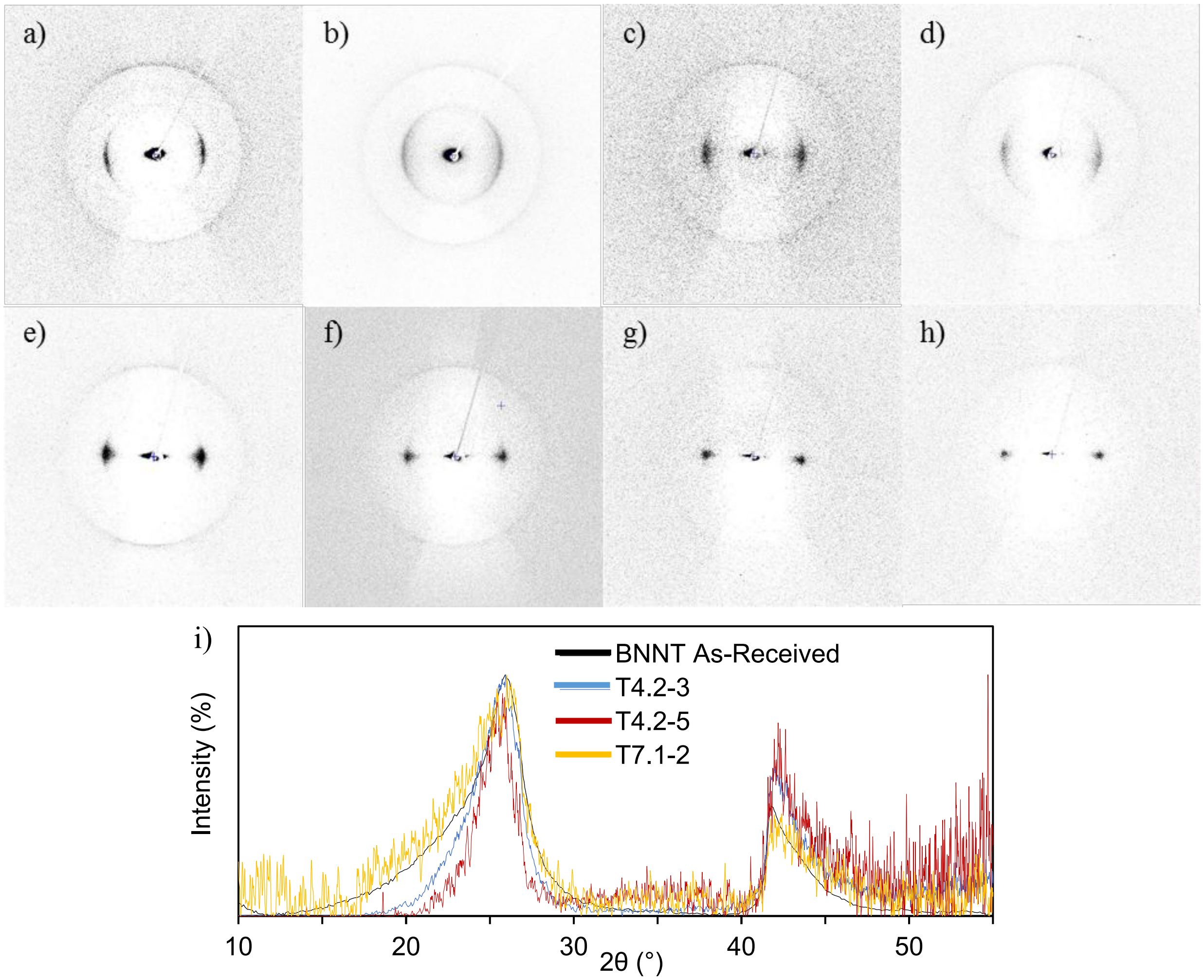

Figure S3: WAXD flat plate photographs of (a) T1.0-1, (b) T2.0-1, (c) T3.0-1, (d) T4.0-1, (e) T4.2-4, (f) T4.2-5, (g) T7.1-2, and (h) T7.1-3; (i) WAXD integrated scan of T4.2-3, T4.2-5, and T7.1-2, as well as that of as-received BNNTs

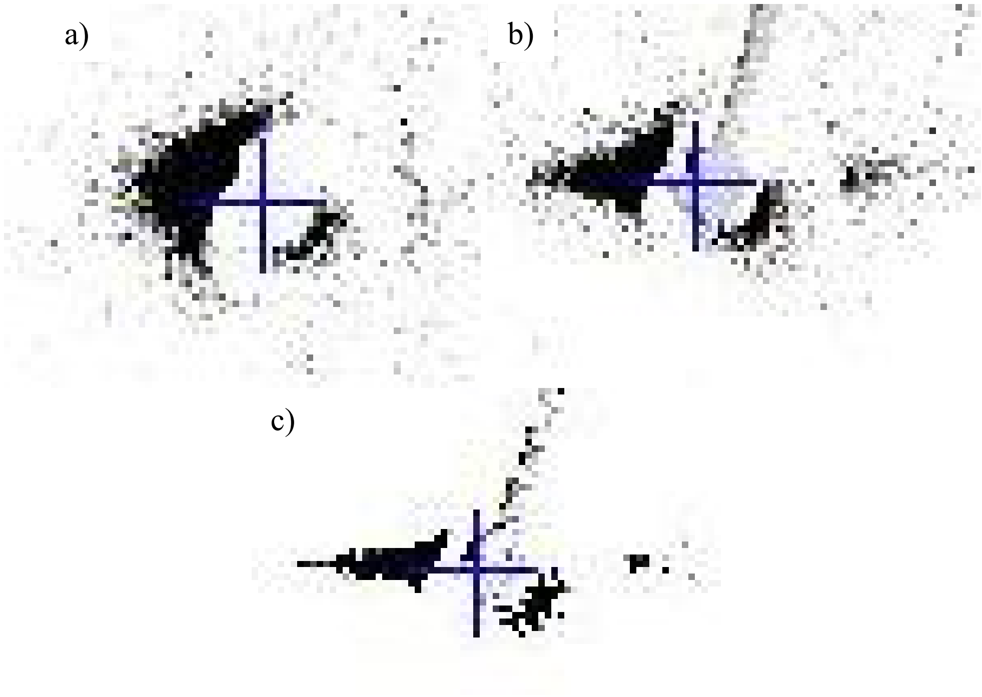

Figure S4: SAXS plate image of a) T4.0-1, b) T4.2-5, and c) T10.2-1

References:

(1) Smith, C. L.; Mintz, K. J.; Gupta, K.; Garg, A.; Wilson, L.; Kumar, S. Processing–structure–property relationships in polymer/boron nitride nanotube composite fibers for electronic packaging applications. *ACS Applied Nano Materials* **2025**, *8*, 18274-18283.
(2) Harrison, H.; Lamb, J. T.; Nowlin, K. S.; Guenthner, A. J.; Ghiassi, K. B.; Kelkar, A. D.; Alston, J. R. Quantification of hexagonal boron nitride impurities in boron nitride nanotubes via FTIR spectroscopy. *Nanoscale Advances* **2019**, *1* (5), 1693-1701.
